# *Babesia bovis* RON2 contains conserved B-cell epitopes that induce an invasion-blocking humoral immune response in immunized cattle

**DOI:** 10.1186/s13071-018-3164-2

**Published:** 2018-11-03

**Authors:** Mario Hidalgo-Ruiz, Carlos E. Suarez, Miguel A. Mercado-Uriostegui, Ruben Hernandez-Ortiz, Juan Alberto Ramos, Edelmira Galindo-Velasco, Gloria León-Ávila, José Manuel Hernández, Juan Mosqueda

**Affiliations:** 1 Immunology and Vaccines Laboratory, C. A. Facultad de Ciencias Naturales, Universidad Autónoma de Querétaro, Carretera a Chichimequillas, Ejido Bolaños, 76140 Queretaro, Queretaro Mexico; 2 Animal Disease Research Unit, USDA-ARS, 3003 ADBF, WSU, P. O. Box 647030, Pullman, WA 99164-6630 USA; 3 CENID-Parasitologia Veterinaria / INIFAP, Carretera federal Cuernavaca-Cuautla #8534, Col. Progreso, 62550 Jiutepec, Morelos Mexico; 4 Facultad de Medicina Veterinaria y Zootecnia, Universidad de Colima, Km. 40 carretera Colima-Manzanillo, 28100 Tecoman, Colima Mexico; 5 Departamento de Zoología, Escuela Nacional de Ciencias Biológicas, Instituto Politécnico Nacional, Carpio y Plan de Ayala, Col. Casco de Santo Tomás, 11340 Mexico City, Mexico; 6 Departamento de Biología Celular, Centro de Investigación y Estudios Avanzados del Instituto Politécnico Nacional, Av. IPN 2508, Col. San Pedro Zacatenco, 07360 Mexico City, Mexico

**Keywords:** Bovine babesiosis, *Babesia bovis*, Tight junction, Invasion process, CLAG domain

## Abstract

**Background:**

*Babesia bovis* belongs to the phylum Apicomplexa and is the major causal agent of bovine babesiosis, the most important veterinary disease transmitted by arthropods. In apicomplexan parasites, the interaction between AMA1 and RON2 is necessary for the invasion process, and it is a target for vaccine development. In *B. bovis*, the existence of AMA1 has already been reported; however, the presence of a homolog of RON2 is unknown. The aim of this study was to characterize RON2 in *B. bovis*.

**Results:**

The *B. bovis ron2* gene has a similar synteny with the orthologous gene in the *B. bigemina* genome. The entire *ron2* gene was sequenced from different *B. bovis* strains showing > 99% similarity at the amino acid and nucleotide level among all the sequences obtained, including the characteristic CLAG domain for cytoadherence in the amino acid sequence, as is described in other Apicomplexa. The *in silico* transcription analysis showed similar levels of transcription between attenuated and virulent *B. bovis* strains, and expression of RON2 was confirmed by western blot in the *B. bovis* T3Bo virulent strain. Four conserved peptides, containing predicted B-cell epitopes in hydrophilic regions of the protein, were designed and chemically synthesized. The humoral immune response generated by the synthetic peptides was characterized in bovines, showing that anti-RON2 antibodies against peptides recognized intraerythrocytic merozoites of *B. bovis*. Only peptides P2 and P3 generated partially neutralizing antibodies that had an inhibitory effect of 28.10% and 21.42%, respectively, on the invasion process of *B. bovis* in bovine erythrocytes. Consistently, this effect is additive since inhibition increased to 42.09% when the antibodies were evaluated together. Finally, P2 and P3 peptides were also recognized by 83.33% and 87.77%, respectively, of naturally infected cattle from endemic areas.

**Conclusions:**

The data support RON2 as a novel *B. bovis* vaccine candidate antigen that contains conserved B-cell epitopes that elicit partially neutralizing antibodies.

**Electronic supplementary material:**

The online version of this article (10.1186/s13071-018-3164-2) contains supplementary material, which is available to authorized users.

## Background

The intraerythrocytic protozoan *Babesia bovis* is the major causal agent of bovine babesiosis, which is one of the most important veterinary diseases transmitted by arthropods. *B. bovis* belongs to the phylum Apicomplexa, which includes *Plasmodium* spp., and *Toxoplasma gondii*, two examples of pathogens within this phylum with medical importance. The parasites of this phylum are characterized by apical organelles such as rhoptries, micronemes and spherical bodies. The proteins related to these organelles are implicated in the invasion and egression of host target cells [1–3]. Importantly, most apicomplexan parasites share four basic steps in the invasion process: (i) attachment to the target host cell; (ii) parasite reorientation to align the apical organelles in close contact with the membrane surface of the target cell; (iii) target cell surface membrane invagination, involving several molecular interactions between protozoan ligands and host receptors so as to make tight junctions; and (iv) parasite internalization, a process that also occurs continuously in the blood of *Babesia* infected bovines. Thus, *B. bovis* merozoites invade red blood cells (RBC), while secreting proteins from the apical organelles and forming close junctions between the membrane of the parasite and the RBC membrane. Once inside the RBC, the parasite multiplies by binary fission in two merozoites, which, upon egression from their original host erythrocyte, go to invade other RBCs to perpetuate this cycle of asexual replication [4–6]. In *Plasmodium falciparum*, the “tight junction” is also known as the “moving junction”, and it was described as a specific and irreversible interaction between two proteins: the apical membrane antigen-1 (AMA-1) located on the merozoite surface and the rhoptry neck protein 2 (RON2), which is integrated to the RBC membrane after its secretion from the rhoptries in a complex formed with other RON proteins [7–9]. The disruption of AMA-1-RON2 interaction ceases the merozoite invasion, making these proteins vaccine candidates [10]. Expression of the neutralization-sensitive AMA-1 was already reported in *B. bovis*; however, there are no previous reports describing the conservation and functional role of RON2 in this parasite despite being recently described in *B. divergens* and *B. microti* [11, 12]. Therefore, the purpose of the present study was to identify a *B. bovis* homolog gene of RON2 and define its pattern of expression and functional relevance.

## Results

### The *B. bovis* genome encodes for a *ron2* orthologous gene

**Fig. 1:**
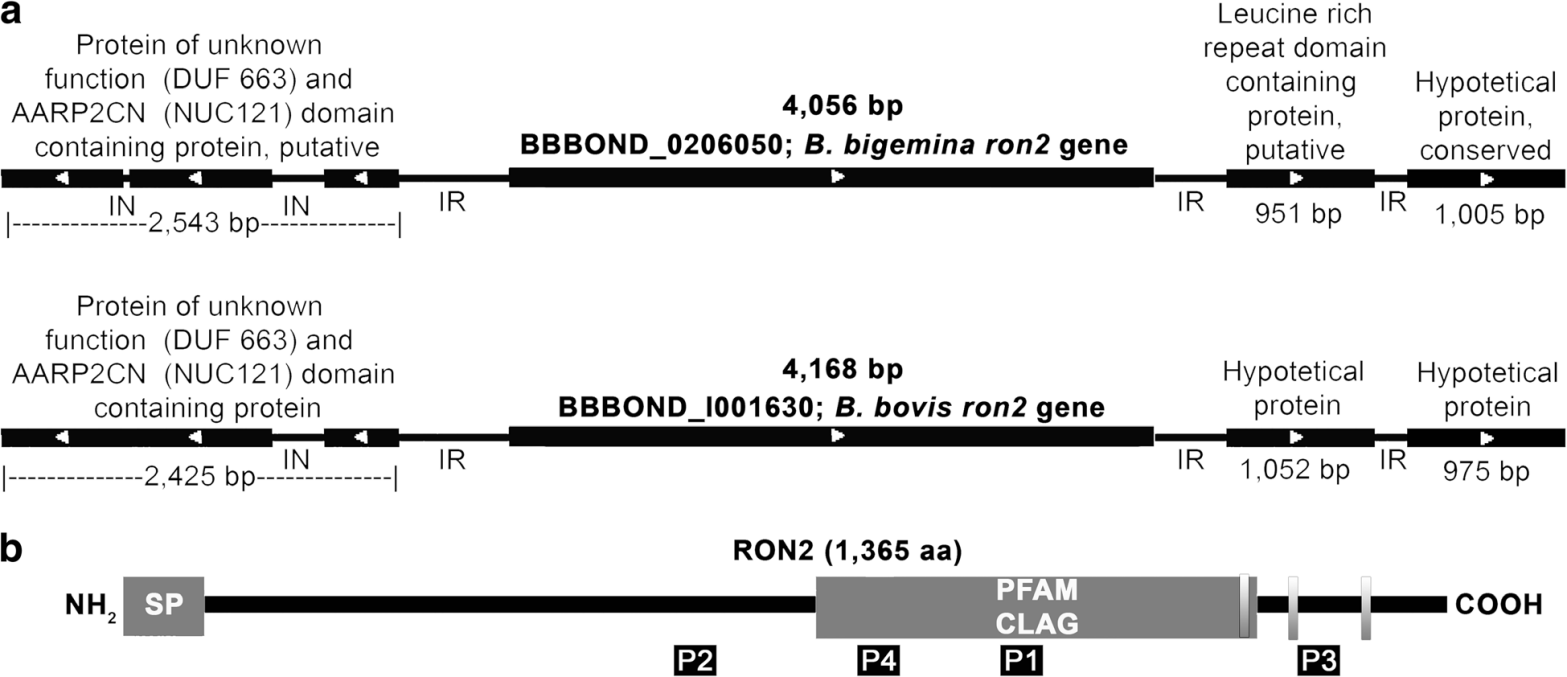
*B. bovis ron2* identification. **a** Synteny map for *B. bovis* and *B. bigemina*. **b** A representation of *B. bovis* RON2, including the signal peptide (SP) located from aa 1 to 19 in different strains of *B. bovis* and the CLAG domain that is located from aa 727 to 1168. Both are depicted as gray boxes*.* Shaded boxes represent the transmembrane regions and black boxes represent the sequences of the selected peptides

A BLAST search against the *B. bovis* T2Bo reference genome using the nucleotide (KU696964.1) and amino acid (AQU42588.1) sequences as a query identified an orthologous gene (BBOV_I001630) in the chromosome 1 contig_1104837696198 (NW_001820854.1). The *ron2* gene does not contain introns and has a very similar synteny between the genome of *B. bovis* and *B. bigemina* (Fig. 1a). The nucleotide sequence has an identity of 70% and the amino acid sequence identity is 64%. Employing eight different pairs of primers (see Table 1), which were designed based using the BBOV_I001630 reference sequence, it was possible to obtain the full sequence of *ron2* in four isolates of *B. bovis*: Chiapas, Colima, Nayarit and Veracruz. The sequence of each isolate was submitted to the GenBank database under the accession numbers MG944401, MG944402, MG944403 and MG944404, respectively. All nucleotide sequences obtained from *ron2* (T2Bo, Chiapas, Colima, Nayarit and Veracruz) have a consensus identity of 99.56%; all RON2 amino acid sequences have a consensus identity of 99.78% and the same predicted physicochemical features: a length of 1365 aa, a signal peptide located in the first 19 aa, a CLAG domain for cytoadherence between the amino acids 176 and 1168, three transmembrane domains, an isoelectric point of 8.9 and a molecular weight of 150 kDa (Fig. 1b and Additional files 1:Table S1, Additional file 2: Table S2, Additional file 3: Table S3). When compared with other species of *Babesia*, RON2 showed an identity of 64% with *B. bigemina* (query cover 98%), 81% with *Babesia* sp. Xinjiang (query cover 73%), 60% with *B. divergens* (query cover 99%), 39% with *B. microti* strain RI (query cover 74%), 42% with the hypothetical protein BEWA 034640 of *Theileria equi* (query cover 89%) and finally 26% with the related Apicomplexa *P. falciparum* (query cover 74%).

**Table 1:** *Babesia bovis ron2* designed primers

Primer	Sequence (5'-3')
Fw0 RON2Bo	CACCTCACCGATATGCGTAC
RvI0 RON2Bo	GAGTCACTGACACCTTGCC
FwI0 RON2Bo	GTCAGTGACTCCCCTCTTCAAG
Rv0 RON2Bo	GGAATCACCGCCTAGTAGC
Fw1 RON2Bo	GCTACTAGGCGGTGATTCC
RvI1 RON2Bo	GCGTCAATAGAGATAAGCAGG
FwI1 RON2Bo	CCTGCTTATCTCTATTGACGC
Rv1 RON2Bo	CAACCCATTGCTTGATTCCC
Fw2 RON2Bo	GGGAATCAAGCAATGGGTTG
RvI2 RON2Bo	CTTTCTTAGCAATAGCGTCGG
FwI2 RON2Bo	CTTCGTTGCTGGAGGCTACATC
Rv2 RON2Bo	CGTTGGATATTCGGTTGAGC
Fw3 RON2Bo	GCTCAACCGAATATCCAACG
Rv3 RON2Bo	CCGTACTTGATTGCTCTGAG
Fw4 RON2Bo	CTCAGAGCAATCAAGTACGG
Rv4 RON2Bo	CACGGATGGCTATGACAATG

*Note*: Eight pairs of primers were designed to get the amplification and sequencing of 4169 bp under the same PCR protocol

### The *ron2* gene is transcribed in *B. bovis* virulent and attenuated strains

**Fig. 2:**
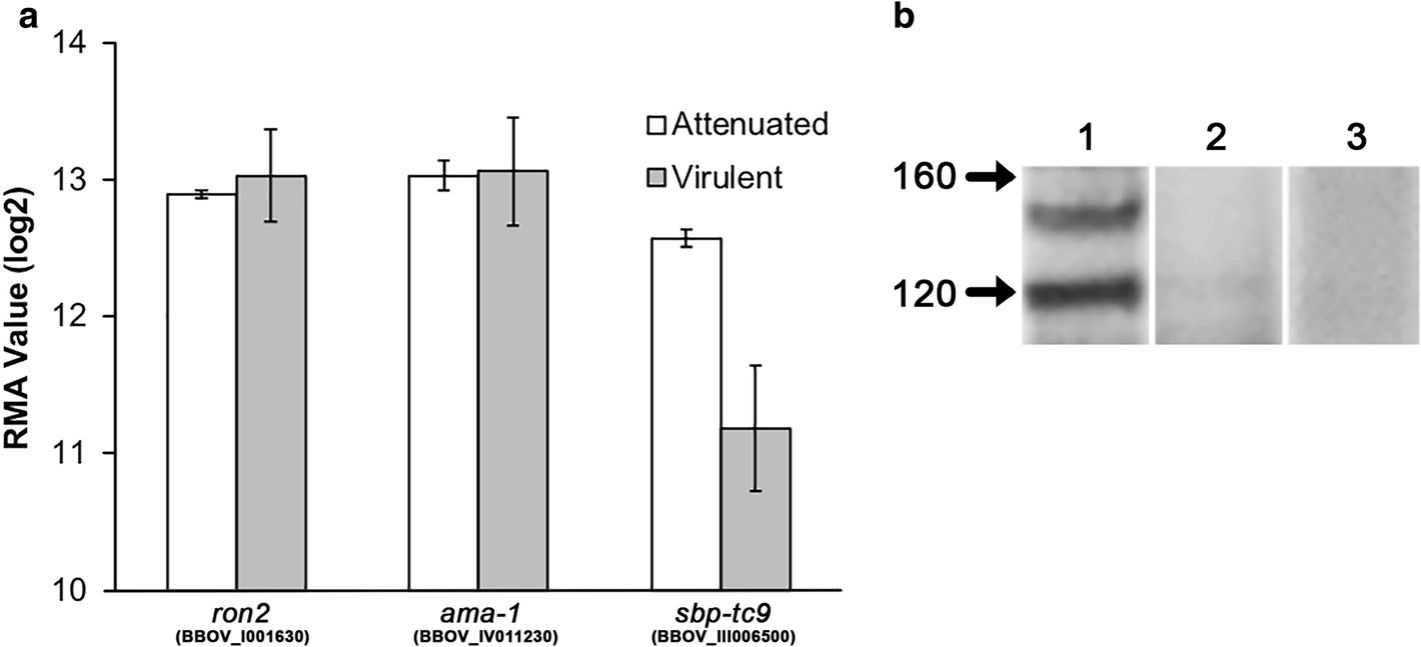
Transcription and expression analysis of *B. bovis ron2*. **a** Bioinformatics transcription analysis. The results on the y-axis are shown by robust multi-array average (RMA) normalized values (log_2_). The comparison of the expression level of *ron2*, *ama-1* and *sbp-tc9* (spherical body protein 2 truncated copy 9) genes between attenuated (white) and virulent (gray) strains is shown on the x-axis. **b** WB expression analysis of RON2. Lane 1: proteins of *B. bovis* iRBC incubated with post-immunization sera anti-RON2; Lane 2: proteins of *B. bovis* iRBC incubated with pre-immunization sera anti-RON2; Lane 3: proteins of nRBC incubated with post-immunization sera anti-RON2. The molecular weight marker is shown in kDa

The *in silico* transcription profiling database available from the whole genome microarray at the PiroplasmaDB portal, shows that the genes *ron2* (BBOV_I001630) and *ama-1* (BBOV_IV011230) of attenuated and virulent strains of *B. bovis* have equivalent transcription levels in both strains, suggesting that the expression levels of *ama-1* and *ron2* genes are similar among both strains (Fig. 2a). To validate the results obtained with this analysis, *sbp-tc9* (spherical body protein truncated copy 9) was also included in the analysis, showing upregulation in the attenuated strain, confirming the results reported previously for this gene [13].

### RON2 is expressed in *B. bovis* and contains immunogenic peptides

To identify conserved, antigenic and immunogenic regions in the RON2 sequence, a bioinformatics strategy was employed. A total of four peptides containing predicted B-cell epitopes were designed (Fig. 1b and Additional file 4: Table S4). The four peptides have the capacity to elicit an immune response, confirming the *in silico* prediction of antigenicity and immunogenicity. The bovine anti-RON2 antisera reacted in western blots with antigens sized between 120 and 160 kDa, which are present only in the iRBC lysate (Fig. 2b, Lane 1). However, these antigens were not recognized by pre-immune sera (Fig. 2b, Lane 2), nor was their reactivity detected with nRBC lysates in immunoblots by anti RON2 antisera (Fig. 2b, Lane 3). The estimated molecular weight of *B. bovis* RON2 is ~150 kDa, which is consistent with the size of the antigen recognized by the anti RON2 peptide antisera. In addition, the immune serum against the selected RON2 peptides reacts with a 120 kDa band present in the sample containing the iRBC lysate (Fig. 2b, Lane 1). These results showed that the bovine anti-peptide antibodies were able to recognize the native *B. bovis* RON2 in immunoblots confirming the antigenicity of the predicted B-cell epitopes included in the synthetic peptides.

### Recognition of *B. bovis* blood stages by anti-RON2 sera

**Fig. 3:**
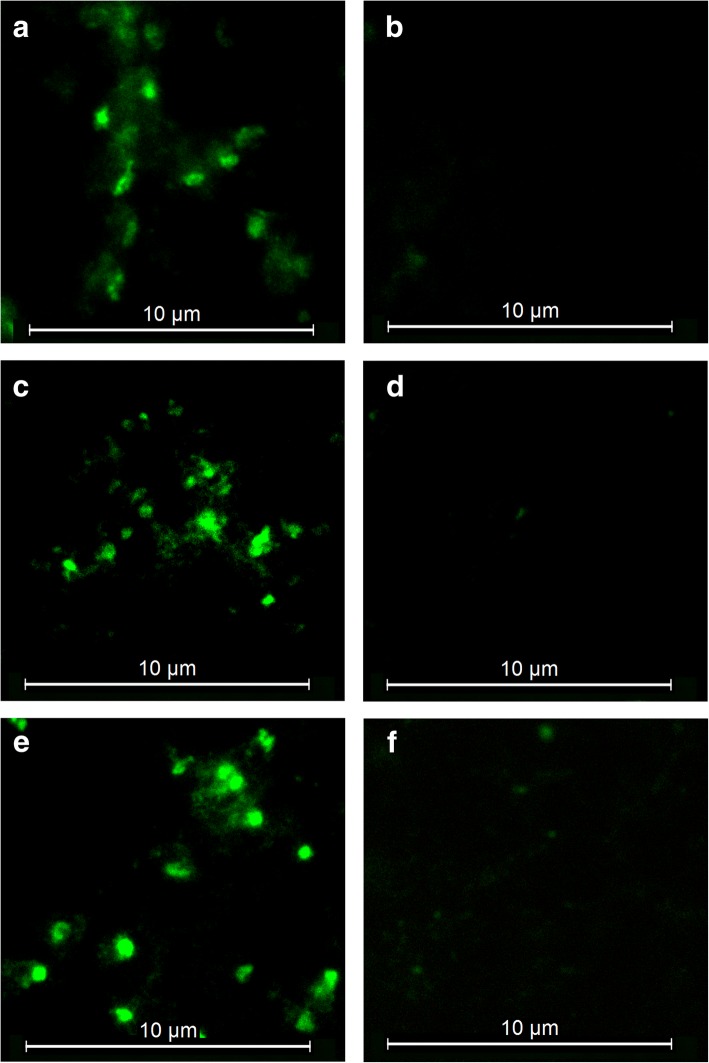
Indirect immunofluorescence of *B. bovis* blood stages detected with antibodies against RON2. Smears of *B. bovis*-infected merozoites were incubated with bovine antiserum against P2 (**a**), P3 (**c**) or serum from naturally infected cattle (**e**). No signal was observed in the pre-immunization serum of cattle immunized with P2 (**b**), P3 (**d**) or cattle immunized only with adjuvant (**f**) used as the negative control. *Scale-bars*: 10 μm

We then analyzed the pattern of reaction of the bovine antibodies against the selected RON2 peptides with *B. bovis* blood stages by IFAT. All the post-immunization sera samples tested showed a very similar staining pattern, consisting of defined and strongly marked dots (Fig. 3a, c). This pattern of reactivity was comparable to the signals observed when smears of *B. bovis*-infected red blood cells were incubated with the serum of naturally infected cattle, employed as a positive control (Fig. 3e). This signal was not observed when *B. bovis*-infected red blood cells were incubated with the pre-immunization sera or with serum from cattle immunized only with adjuvant (Fig. 3b, d, f). Additionally, it was verified by confocal microscopy, that the observed signal corresponds to intraerythrocytic merozoites (see Additional file 5: Figure S1).

### *In vitro* neutralization assay

**Fig. 4:**
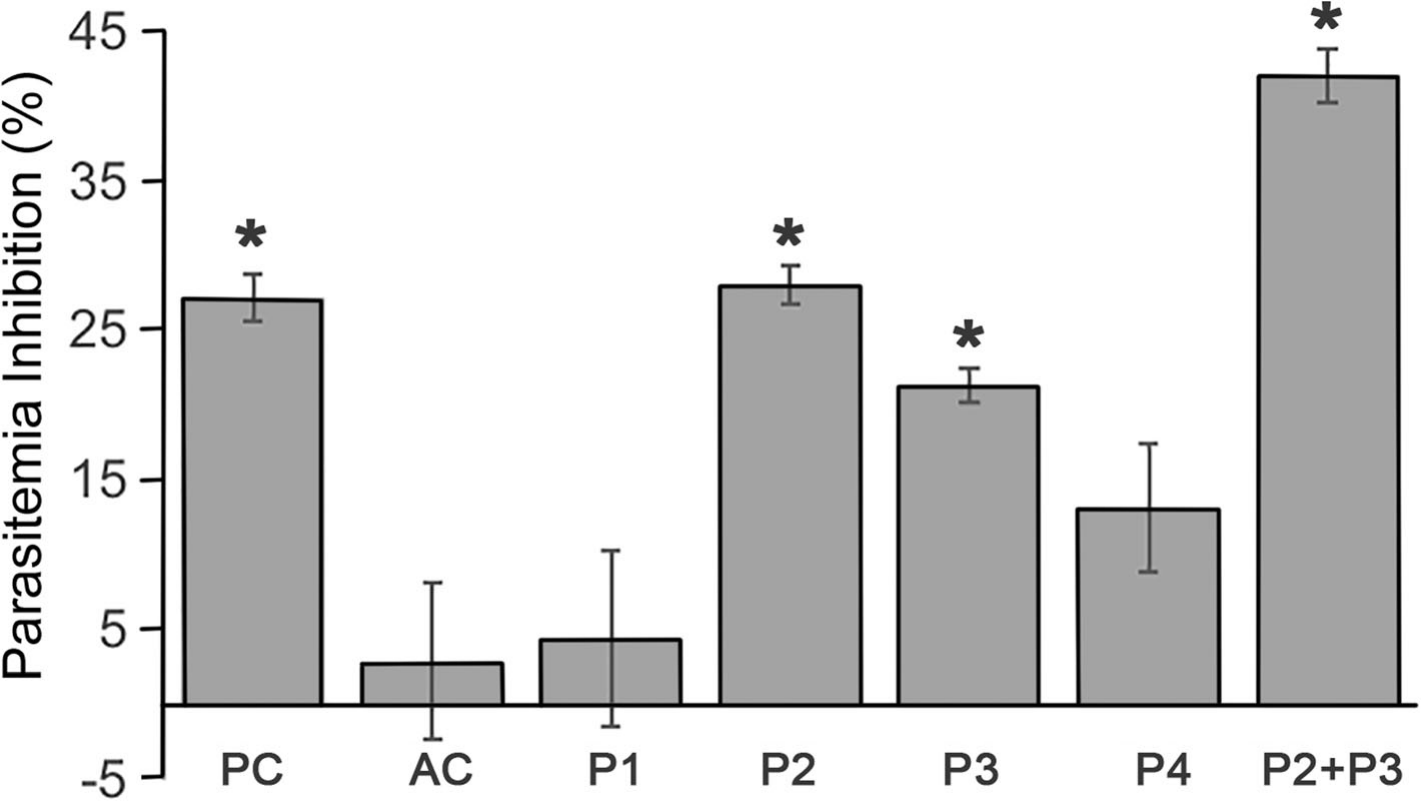
Neutralization assay. The results on the y-axis are shown as percentages of the parasitemia inhibition. The evaluation of the inhibition generated by different antibodies is shown on the x-axis. *Abbreviations*: PC, positive control; AC, adjuvant control (PBS + ADJ); P1, anti-Peptide1 antibodies; P2, anti-Peptide2 antibodies; P3, anti-Peptide3 antibodies; P4, anti-Peptide4 antibodies; P2+P3, a mix of the anti-Peptide2 and anti-Peptide3 antibodies. There were significant differences between the pre- and post-immunization serum samples (*P < 0.05)

We then tested the capacity of anti-RON2 antibodies to block merozoite invasion in an *in vitro* neutralization assay. The results, shown in Fig. 4, demonstrate a statistically significant difference in the percentage of parasitemia between the culture supplemented with the post-immunization serum and the culture supplemented with the pre-immunization serum only for P2 and P3 (P2: *t*_(4)_= 19.81, *P* < 0.0001; P3: *t*_(4)_= 33.64, *P* < 0.0008). In addition, combination of the anti-P2 and P3 anti-sera in the *in vitro* neutralization assay resulted in a significant (*t*_(4)_= -10.6, *P* < 0.0004) increase of parasite inhibition (42.09%).

### Neutralization-sensitive peptides are implicated in a natural immune response

We used an indirect ELISA to evaluate whether antibodies in 90 serum samples from cattle naturally infected with *B. bovis* developed antibodies against the neutralization-sensitive epitopes present in the selected RON2 peptides. The results are shown in Table 2: peptide 2 was recognized by 75 sera (83.33%), and peptide 3 was recognized by 79 sera (87.77%). Thus, both peptides were recognized by all positive controls and the 11 negative serum samples analyzed did not recognize any of the peptides evaluated.

**Table 2:** RON2 peptides are recognized by sera of naturally infected cattle

State	Farm	Peptide 2			Peptide 3		
		Positive	Negative	Total	Positive	Negative	Total
Jalisco	A	6	1	12	6	1	12
	B	2	0		2	0	
	C	3	0		2	1	
Veracruz	A	20	3	36	18	5	36
	B	10	2		11	1	
	C	1	0		1	0	
Querétaro	A	1	1	12	2	0	12
	B	1	0		1	0	
	C	2	1		3	0	
	D	1	0		1	0	
	E	3	1		4	0	
	F	1	0		1	0	
Guanajuato	A	2	0	2	2	0	2
Chiapas	A	1	0	7	1	0	7
	B	6	0		5	1	
Chihuahua	A	1	2	16	3	0	16
	B	1	0		1	0	
	C	2	1		3	0	
	D	1	0		1	0	
	E	6	2		7	1	
Sinaloa	A	1	0	5	1	0	5
	B	0	1		1	0	
	C	3	0		2	1	
Total positive/negative		75	15		79	11	

*Key*: Positive, *B. bovis* positive serum samples that recognized the peptide; Negative, *B. bovis* positive serum samples that did not recognize the peptide

## Discussion

In the present study we demonstrated the conservation of a single copy gene encoding for RON2 in the *Babesia bovis* genome, which is highly conserved among four distinct strains isolated in Mexico and the reference strain T2Bo. The synteny of *ron2* between *B. bovis* and *B. bigemina*, as well as the high identity of this gene among all the compared species, suggest an implication of RON2 in a well conserved, likely essential, parasite function in the genus *Babesia*, which is also maintained in other related Apicomplexa parasites through the presence of the CLAG domain. Importantly, the implication of this domain in cytoadherence during the erythrocyte invasion process has been previously demonstrated in *P. falciparum* [14, 15]. Additionally, the transcription analysis showed a similar level of transcripts in both attenuated and virulent strains, implying that the levels of expression of this gene are not critical for the modulation of a virulent phenotype of *B. bovis*, as previously demonstrated for the *sbp2t-11* gene [16]. Furthermore, the transcription level of *ron2* was similar to that observed for *ama-1*, a gene involved in the same invasion step, as is described in other apicomplexan parasites [17]. Taking together, the data showing similarity between the level of expression of these proteins among virulent and attenuated strains may support the notion that the mechanism of invasion is highly conserved because it is required for the maintenance of the parasite inside the host. Moreover, similar levels of expression of the *ama-1* and *ron2* genes suggests a concerted function among these two proteins in *Babesia* parasites, as already described for *Plasmodium*. Since AMA-1 was also shown to be neutralization sensitive [11], this would support the evaluation of these two proteins in a single vaccine formulation. Specific bovine anti-RON2 antibodies were produced and used to detect RON2 in *B. bovis* protein extracts, showing a band of approximately 150 kDa in the western blot, which correlates with the expected molecular weight of the protein. Additionally, a band of a lower molecular weight was observed in the same protein extracts but was not present in the extracts of uninfected erythrocytes. Since the peptide used to generate the RON2 antibodies was analyzed by bioinformatics in the *B. bovis* genome and proved to be specific to RON2, the *in silico* analysis supports that this band is not another protein from the parasite, suggesting that this lower band could be generated due to a RON2 proteolysis process. This proteolysis event on RON2 has also been observed in other *Babesia* species [12].

Although peptide design remains a challenge due to paradoxically inconsistent results obtained with bioinformatics tools [18], in the present study we successfully designed four immunogenic peptides that generated antibodies in immunized bovines and that were able to recognize blood stage parasites. The anti-RON2 antibodies were also able to identify the native protein expressed in blood stage parasites demonstrated by IFAT and confocal microscopy. This is significant as the selected B-cell epitopes used for the generation of these antibodies were predicted on the linear sequence of RON2, suggesting that this part of the sequence is accessible to antibodies in the native protein. It has been demonstrated that RON2 localizes specifically to the neck region of the rhoptries, which possess a protein repertoire conserved across the phylum and involved in tight-junction formation [17]. Co-localization or electron microscopy studies are necessary to determine the exact subcellular location of RON2 in *B. bovis*.

Interestingly, only antibodies produced by peptides P2 and P3 were able to block the invasion process, as observed in the *in vitro* neutralization assays. A higher inhibition of parasitemia was observed when antibodies anti-P2 and anti-P3 were used together, suggesting a synergic effect. This type of synergy can be used to improve the inhibition effect, for example by mixing antibodies against other proteins, but it also demonstrates that the antibodies can act independently in their inhibitory activity, and no allosteric inhibitory effects are present. In *Plasmodium yoelii*, for example, it was described that a mix of antibodies against a complex of AMA-1 and RON2L completely inhibited the invasion process through the disruption of the specific interaction between these two peptides, and this complex elicited protective immunity in *Aotus* monkeys against a virulent strain of *P. falciparum* [19, 20]. RON2L is a peptide located between the second and third predicted transmembrane region in *P. falciparum* RON2. This location is the same for the P3 designed in this study, highlighting the importance of this peptide as a blocking vaccine candidate. These data demonstrate that targeting epitopes in RON2 with specific antibodies significantly impairs the ability of the parasite to invade erythrocytes. Importantly, the effect is additive, suggesting that the epitopes recognized at least by antibodies reactive with two predicted B-cell epitopes work independently and are accessible to interact with the antigen. The data is also strongly suggestive of a possible role of RON2 in erythrocyte invasion.

Finally, we demonstrated that RON2 generates antibodies in cattle naturally infected with *B. bovis*. These findings indicate that the predicted B-cell epitopes contained in the peptides have a role in the humoral immune response under natural conditions and that these B-cell epitopes are conserved among strains from different geographical locations, which have been reported to have high antigenic variation [21, 22], demonstrating that the selected neutralization-sensitive RON2 peptides are implicated in immune responses in bovines naturally infected with *B. bovis.* Another important implication emerging from these results is that the antibodies against RON2 epitopes may help the chronically infected animals to prevent emergence of clinical signs on the face of parasite persistence, suggesting a direct role of RON2 in the development of protective immunity, and supporting the use of this antigen as a potential vaccine candidate. Although we presented evidence of the conservation of RON2 in different Mexican strains of *B. bovis* and showed the biological effect of conserved anti-RON2 antibodies against a USA strain (T3Bo), and the identity between the amino acid sequences is very high (99.78%), the fact that 15 sera for peptide 2 and 11 sera for peptide 3 did not recognize the respective peptides (out of 90 in total), suggests that some degree of variation may occur between strains. It is, therefore, necessary to evaluate a worldwide conservation of RON2 by obtaining sequences or testing sera samples from other countries. The fact that antibodies against two RON2 peptides were unable to completely block the parasite growth *in vitro*, suggests that antibodies against peptides from additional antigens are necessary for vaccine development against this pathogen.

## Conclusions

In summary, *ron2* is a functional gene in *B. bovis* that codes for a protein with a characteristic CLAG domain. RON2 has highly conserved B-cell epitopes that elicit neutralizing antibodies in bovines and are recognized by protective antibodies in naturally infected cattle. All these data together emphasize the importance of RON2 as a vaccine candidate to prevent bovine babesiosis.

## Methods

### *Babesia bovis* DNA extraction from field isolates

*Rhipicephalus* (*Boophilus*) *microplus* ticks were manually collected from bovines from four different states in Mexico: Chiapas, Colima, Nayarit and Veracruz. They were individually incubated for oviposition and then tested for *Babesia* spp. infection by microscopic examination of hemolymph for kinete detection [23]. DNA was purified from the infected ticks [24] and was used for specific nPCR-diagnosis of *B. bovis* [25]. Larvae from ticks infected only with *B. bovis* were used to infest one splenectomized calf for each isolate. Additionally, each calf was treated with an acaricide five days after infestation to avoid transmission of *B. bigemina* by infected nymphs and the parasitemia was monitored daily by examination of blood smears stained with Giemsa. When the parasitemia exceeded 1%, infected blood from the jugular vein was collected in transfusion bags with ACD anticoagulant solution (375 Blorecep, Industrias Plasticas Medicas, Ayala, Morelos, Mexico). Genomic DNA was extracted using the illustra blood genomicPrep Mini Spin kit (GE Healthcare, Piscataway, NJ, USA) and was stored at -20 °C.

### Identification and sequencing of *ron2* in *Babesia bovis*

The *B. bigemina ron2* nucleotide (KU696964.1) and amino acid (AQU42588.1) sequences were used as a query in a BLAST search of the SANGER institute database [26] against the *B. bovis* T2Bo reference genome. The synteny of the chromosomal region of the *ron2* gene was determined and compared between *B. bovis* and *B. bigemina* through PiroplasmaDB at the EuPathDB portal [27, 28]. Eight pairs of primers (see Table 1) were designed based on the *B. bovis* T2Bo *ron2* gene using Oligoanalyzer 3.1 [29]. These primers were designed to amplify the whole *ron2* sequence using a common PCR protocol: an initial denaturation at 94 °C for 4 min, followed by 30 cycles of denaturation at 94 °C for 30 s, annealing at 56 °C for 45 s, and extension at 72 °C for 45 s, followed by a final extension at 72 °C for 7 min. DNA from four field isolates of *B. bovis*, each from a different state in Mexico (Chiapas, Colima, Nayarit and Veracruz), were used for the amplification and sequencing of 4169 bp of the *B. bovis ron2* gene. All of the amplifications were cloned into a pCR™ 4-TOPO® vector using a TOPO® TA Cloning® kit (Invitrogen, Carlsbad, CA, USA), and *E. coli* strain TOP10 cells were transformed with the vector following the manufacturer’s instructions (Invitrogen). For each amplification, two positive colonies determined by PCR were selected to be sequenced with the dideoxy chain-termination method by the Biotechnology Institute of Universidad Nacional Autonoma de Mexico (UNAM, Cuernavaca, Mexico). The assembly and obtaining of consensus sequences was undertaken using BioEdit 7.2.6 and CLC Genomics Workbench 7.5.

The sequences obtained were compared against *B. bigemina* (AQU42588.1), *Babesia* sp. Xinjiang (ORM40446.1), *B. divergens* (ADM34975.2), *B. microti* strain RI (XP_021338832.1), *Theileria equi* (XP_004830272.1) and *P. falciparum* (BAH22613.1). The *B. bovis* sequences were analyzed with bioinformatics programs to: (i) identify open reading frames using the ORF finder program [30]; (ii) determine the signal peptide with the programs SignalP 4.0 [31] and SMART [32]; (iii) find conserved domains and their localization with SMART [32]; (iv) assess the presence of transmembrane helices using the TMHMM program [33]; and (v) determine the isoelectric point and the molecular weight using CLC Genomics Workbench 7.5.

### *In silico* transcription analysis

RMA values from transcriptomic analyses of biological replicate (BR) sample pairs were obtained from previously published data deposited in PiroplasmaDB in the EuPathDB portal [27]. The transcription of the *ron2* gene (BBOV_I001630) was evaluated among the attenuated and virulent strains, and the level of transcription of the *ama-1* gene (BBOV_IV011230) was also evaluated. These results were validated with the transcript expression level of the *spherical body protein 2 truncated copy 9* gene (*sbp2-tc9*, BBOV_III006500). This analysis was performed in the PiroplasmaDB portal [13, 27].

### Expression analysis

To produce anti-RON2 antibodies, a conserved region among all the isolates was analyzed to find predicted linear B-cell epitopes using different programs: ABCpred [34], BCEpred [35] and antibody epitope prediction IEDB-AR [36]. The conserved regions were located with multiple sequence alignments using Clustal Omega [37]. The peptide was synthesized in a Multiple Antigen Peptide System of 8 branches (MAPS-8) by GL Biochem (Shanghai, China). The synthetic peptide (see Additional file 4: Table S4) was solubilized in PBS (pH 7.4) and emulsified v/v with the adjuvant Montanide ISA 71vg (Seppic, Puteaux, Paris, France) at a final concentration of 100 μg/ml. Finally, two cattle born in a tick-free area and free of antibodies against *B. bovis* and *B. bigemina* by IFAT were immunized four times at 21-day intervals with the peptide/adjuvant emulsions described above. Additionally, two control cattle were immunized just with the same adjuvant emulsified with PBS (1:1) under identical conditions. The immunizations were performed *via* subcutaneous injection in the scapular region with 1 ml of the corresponding emulsion mixes in each bovine. Sera samples were collected from each animal before the first immunization and 15 days after the last immunization.

The bovine sera containing specific anti-RON2 antibodies and control sera were diluted at 1:20 with PBS containing non-infected erythrocyte (nRBC) lysate and 5% skim milk to evaluate the expression of RON2 in a western blot analysis (WB). Briefly, *Babesia bovis*-infected erythrocytes (iRBC) were obtained from an *in vitro* culture with 42% parasitemia; lysates were prepared by washing the cells several times with ice-cold PBS containing protease inhibitor (Roche-Applied Science, Penzberg, Upper Bavaria, Germany) until the supernatant was clear. Then, the pellet was frozen at -80 °C, thawed on ice and washed again three times. All the centrifugations between washes were performed at 2500× *g* for 10 min. After washes, the parasite pellet was suspended in 2× lysis buffer v/v (100 mM Tris, 10 mM EDTA, 2% NP-40) containing protease inhibitor (Roche-Applied Science). The suspended pellet was kept at -80 °C until used. Finally, when the pellet was thawed, it was maintained on ice; the loading buffer was then added and the sample sonicated and then centrifuged briefly. The supernatant was used in an SDS-PAGE (4–20%) and was run at 100 V for 1 h employing Mini-PROTEAN TGX precast gels (Bio-Rad Laboratories, Richmond, CA, USA). The proteins were transferred to a nitrocellulose membrane at 100 V, for 1 h. The membrane was blocked overnight at 4 °C with PBS containing 5% skim milk (PBS-M). The membrane was incubated for 1 h at room temperature with each diluted antiserum and washed five times with agitation at room temperature in PBS and 0.1% Tween (PBS-T). The membrane was incubated under the same conditions employing a donkey anti-bovine IgG antibody conjugated with HRP (Jackson ImmunoResearch, West Grove, PA, USA), diluted at 1:500 in TBS-T (0.1%), and followed by a final wash. Finally, the reaction was visualized with LumiFlash Prime Chemiluminescent Spray (Visual Protein, Taiwan, China).

### Generation of anti-RON2 antibodies

Four peptides (see Additional file 4: Table S4) were designed and each peptide was emulsified and immunized into two bovines using the methodology described above. Pre- and post-immunization sera samples were collected and analyzed for the presence of antibodies against the specific RON2 peptide by an indirect ELISA test as it was described elsewhere [38]. Briefly, each serum sample was added at a dilution of 1:3000 in PBS, and the secondary antibody, a donkey anti-bovine IgG antibody conjugated with HRP (Jackson ImmunoResearch), was added at 100 μl/well, diluted 1:500 in PBS. The reaction was detected in an iMark™ Microplate Absorbance Reader (Bio-Rad Laboratories) at 450 nm and analyzed with Microplate Manager 6 software (Bio-Rad Laboratories). All samples were tested in triplicate, and the cut-off values were determined as the average of the pre-immunization OD value for each bovine plus 3 standard deviations.

### Parasite recognition by anti-RON2 antibodies using an indirect immunofluorescence antibody test (IFAT)

To determine if the anti-RON2 antibodies against each peptide recognize the native protein in the parasite, an indirect immunofluorescence antibody test (IFAT) was performed [39]. Smears of bovine blood infected with *B. bovis* (Chiapas isolate) were permeated at 4 °C for 15 min with acetone (90%) diluted in ethanol. The bovine RON2 antisera were tested at a dilution of 1:100 and detected with Alexa Fluor-488 conjugated with Protein G (Molecular Probes®, Eugene, OR, USA). All the incubations were performed at 37 °C in a humidity chamber for 1 h. Between each incubation, three washes were performed in PBS-T (0.1% Tween20); each wash was done for 5 min with agitation, with the final step in distilled water as described elsewhere [39]. Serum from a bovine naturally infected with *B. bovis* was used as a positive control, and the serum of a bovine immunized with PBS and adjuvant was used as a negative control.

### Neutralization assay

To determine if the invasion process could be blocked by the anti-RON2 antibodies, a neutralization assay (NA) was carried out as previously described [40, 41], using an *in vitro* culture of *B. bovis*. The *B. bovis* T3Bo strain (provided by the ADRU-USDA lab at Washington State University) was cultured in a 96-well plate using 200 μl per well with 5% hematocrit. First, an incubation step was done in an atmosphere of 5% CO_2_ for 30 min at 37 °C with a mix containing 60% HL-1 medium (pH 7.2), 40% sera and 1% iRBC with 1% parasitemia. Then, culture medium with 4% nRBC was added and, after a gentle mix, 200 μl of sample was split into three wells. The culture was maintained at 37 °C in a 5% CO_2_ atmosphere for 72 h with changes of media (120 μl media plus 30 μl sera) every 24 h. Serum from a non-infected steer (c1537) born in a tick-free area was used as a negative control (NC) and the serum from a steer (C168) inoculated with *B. bovis* T2Bo and challenged with *B. bovis* T3Bo was used as positive control (PC). As a control for the effect of the adjuvant (AC), pre- and post-immunization serum from a heifer immunized only with PBS plus adjuvant was used. At the end of the incubation, the percentage of parasitized erythrocytes (PPE) was determined by flow cytometry [42]. For statistical analysis, an independent Student’s t-test was used, where *P*-values < 0.05 were considered significant. The percentages of parasitemia inhibition (% pi) for the anti-RON2 antibodies and the AC were calculated with the following formula: % pi = 100 – ([(PPE Post) / (PPE Pre)] × 100). The formula for the PC was: % pi = 100 – ([(PPE PC) / (PPE NC)] × 100).

### *Babesia bovis* RON2 recognition by naturally infected bovines

A total of 112 bovine serum samples were tested against peptides P2 and P3 using the indirect ELISA protocol described above: all of the 90 serum samples collected from cattle living in regions of Mexico where babesiosis is endemic were positive to *B. bovis* infection as determined by IFAT; 11 bovine serum samples previously confirmed positive to *B. bovis* antibodies, were used as positive controls. Eleven serum samples were from cattle born and maintained in a tick-free area and they were used as negative controls. All serum samples were tested in triplicate at a 1:50 dilution in PBS and incubated for 1 h at 37 °C. As a secondary antibody, a goat anti-bovine IgG (H+L) conjugated with HRP (Jackson ImmunoResearch) was used at a 1:1500 dilution in PBS. The ELISA plates were incubated for 1 h at 37 °C. The cut-off value was determined by adding the average of the negative control OD values plus 3 standard deviations.

## Additional files

Additional file 1: **Table S1.** Percent nucleotide identity matrix. Comparison of the *ron2* nucleotide sequences obtained. (XLSX 9 kb)

Additional file 2: **Table S2.** Percent amino acid identity matrix. Comparison of the RON2 amino acid sequences obtained. (XLSX 9 kb)

Additional file 3: **Table S3.** RON2 physicochemical features. (XLSX 10 kb)

Additional file 4: **Table S4.** Peptides designed in conserved regions with predicted B-cell epitopes. Sequence of the peptides designed, length and position in the RON2 amino acid sequence. (XLSX 9 kb)

Additional file 5: **Figure S1.** Intraerythrocytic *Babesia bovis* merozoites express RON2. Merozoites were incubated with bovine antiserum against RON2 (**a**-**c**), bovine pre-immune serum (**d-f**), or bovine antiserum against *B. bovis* (**g**-**i**), then with an Alexa Fluor-488 conjugated with protein G (green fluorescence) and DAPI for DNA staining (blue fluorescence). The smears were analyzed by confocal microscopy using the following channels: individual channel for Alexa Fluor-488 (**a**, **d** and **g**), individual channel for DAPI (**b**, **e** and **h**) or merged channels for Alexa Fluor-488 and DAPI (**c**, **f** and **i**). *Scale-bars*: 10 μm. (TIF 17380 kb)

## Abbreviations

nRBC: non-infected erythrocyte
iRBC: *Babesia bovis*-infected erythrocytes
NC: Negative control
PC: Positive control
AC: Adjuvant control
PPE: Percentage of parasitized erythrocytes
% pi: Percentages of parasitemia inhibition
