# Supplementary figures and images for: Babesia bovis RON2 contains conserved B-cell epitopes that induce an invasion-blocking humoral immune response in immunized cattle
Source: Parasit Vectors. 2018 Nov 3;11:575. doi: 10.1186/s13071-018-3164-2 (PMC6215676; doi:10.1186/s13071-018-3164-2)

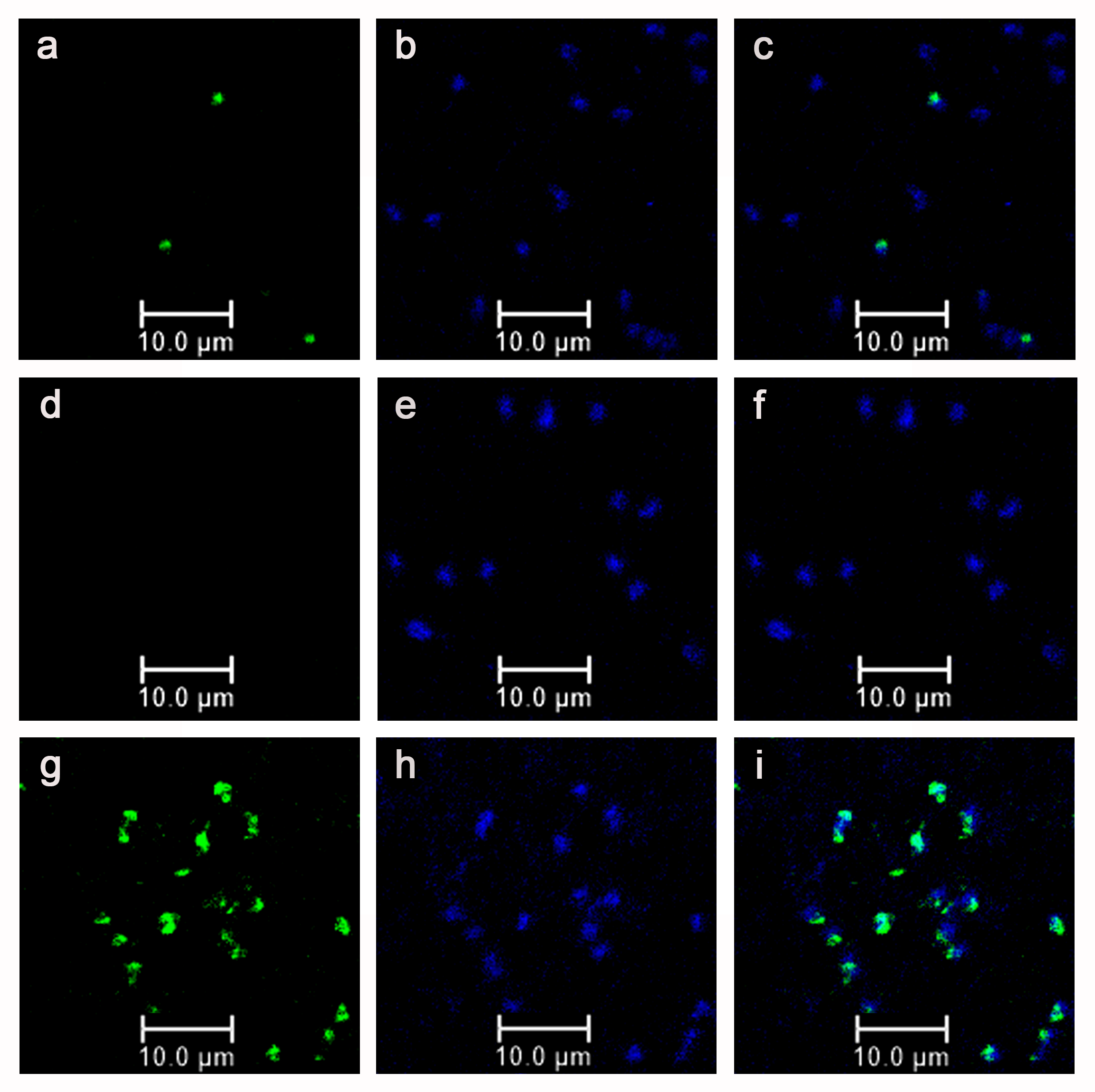

Supplement: Supplementary file 5 — Figure S1. Intraerythrocytic Babesia bovis merozoites express RON2. Merozoites were incubated with bovine antiserum against RON2 (a-c), bovine pre-immune serum (d-f), or bovine antiserum against B. bovis (g-i), then with an Alexa Fluor-488 conjugated with protein G (green fluorescence) and DAPI for DNA staining (blue fluorescence). The smears were analyzed by confocal microscopy using the following channels: individual channel for Alexa Fluor-488 (a, d and g), individual channel for DAPI (b, e and h) or merged channels for Alexa Fluor-488 and DAPI (c, f and i). Scale-bars: 10 μm. (TIF 17380 kb) [file 13071_2018_3164_MOESM5_ESM.tif]
